# Effect of different types of Tai Chi exercise programs on the rate of change in bone mineral density in middle-aged adults at risk of osteoporosis: a randomized controlled trial

**DOI:** 10.1186/s13018-023-04324-0

**Published:** 2023-12-11

**Authors:** Jianda Kong, Chunlan Tian, Lei Zhu

**Affiliations:** College of Sports Science, Qufu Normal University, Qufu, China

**Keywords:** Tai Chi, 24-Style TC Chuan, TC Kung Fu Fan, TC Softball, Middle-aged and elderly, Bone mineral density, Osteoporosis

## Abstract

**Objective:**

To evaluate three Tai Chi (TC) exercise programs as intervention measures to compare their effects on improving rate of change in bone mineral density (BMD) in elderly individuals with osteoporosis (OP) and to propose the optimal exercise duration.

**Methods:**

A randomized controlled trial (RCT) was conducted to identify study participants based on inclusion and exclusion criteria. Due to subject attrition, the number of participants analyzed decreased from 60 to 49. These participants were divided into four groups: 24-style TC Chuan group (24TCCG) (*n* = 13, 7 males/6 females), TC Kung Fu Fan group (TCKFFG) (*n* = 12, 5 males/7 females), TC Softball group (TCSBG) (*n* = 11, 6 males/5 females), and a control group (CG) (*n* = 13, 6 males/7 females). Except for the control group, each group received different TC exercise programs four times a week for 60 min per session, lasting for 16 weeks. BMD was measured using dual-energy X-ray absorptiometry (DXA) at the L2-L4 lumbar vertebrae, Ward's triangle, femoral neck, and greater trochanter. The rate of change of BMD was calculated using the formula.

**Results:**

Compared with CG, all three TC groups showed significant improvements in BMD changes (*P* < 0.05), but their effects on the improvement of femoral neck and greater tuberosity BMD change rates were similar (*P* > 0.05). In addition, compared to the other exercise regimens, 24TCCG demonstrated more significant improvements in BMD at the L2-L4 lumbar vertebrae region and exhibited a more pronounced improvement in Ward's triangle BMD after only 8 weeks (*P* < 0.05). Short-term (≤ 4 weeks) TCKFFG was more effective than TCSBG in enhancing femoral neck BMD (*P* < 0.05). However, statistical significance was not found (*P* > 0.05) in all other cases.

**Conclusion:**

These three TC exercise programs have similar positive effects on the BMD of the femoral neck and greater trochanter. However, compared with other exercise schemes, 24TCC showed a more significant improvement in BMD of the L2-L4 lumbar vertebrae region after just 8 weeks, as well as a more pronounced improvement in BMD of Ward's triangle. In terms of improving femoral neck BMD, TCKFF was found to be more effective than TCSB in less than 4 weeks. This study provides evidence for the effectiveness of TC exercise in improving BMD and preventing OP in the middle-aged and elderly high-risk population.

**Supplementary Information:**

The online version contains supplementary material available at 10.1186/s13018-023-04324-0.

## Introduction

Aging is associated with decreased bone health and an increased risk of osteoporosis (OP) [1, 2]. OP is a degenerative disease characterized by a decrease in bone mineral density (BMD) and an increased susceptibility to fractures [3, 4]. Fractures caused by OP are becoming increasingly common in women over the age of 55 and men over the age of 65, leading to severe bone-related conditions [2, 5]. Evidence suggests that drug treatment has some effect in relieving OP, but the effectiveness and safety of this approach are still limited [6–8]. Therefore, effective interventions are needed to prevent and mitigate the risk of bone loss in middle-aged and older populations [9]. Evidences suggest that high-load skeletal muscle stimulation or high-intensity exercise has been shown to increase bone mineral content and enhance BMD in middle-aged and older populations [10–13].

In recent years, there has been growing interest in the potential benefits of Tai Chi (TC) exercise in improving BMD in this population. TC is a unique Chinese exercise method that combines gentle, flowing movements with rhythmic breathing and meditation [14]. It is considered a safe, affordable, and effective form of exercise that promotes physical well-being [14]. Previous studies have shown that TC exercise can improve balance, reduce falls, and have a positive impact on bone density [15, 16]. However, existing research on the effectiveness of TC exercise in preventing OP in middle-aged and older populations has yielded inconsistent results [13, 17–19], and most of these studies have focused on healthy individuals or patients already diagnosed with OP, with limited attention given to high-risk populations [20, 21]. Additionally, TC exercises encompass various programs such as 24-style TC Chuan (24TCC), TC Kung Fu Fan (TCKFF), and TC Soft Ball (TCSB). However, there is currently no evidence suggesting differences in the preventive and therapeutic effects of different types of TC exercise programs on OP in the middle-aged and elderly population. Therefore, our understanding of the comparative efficacy of different TC exercise protocols in improving BMD is limited [18]. This highlights the importance of investigating the impact of different TC exercise programs on BMD in high-risk groups of OP among middle-aged and elderly individuals. Furthermore, the existing evidence-based medicine research on TC exercises for BMD improvement mainly focuses on the intervention of the 24TCC program [13, 17–21]; thus, research on other TC exercise programs is relatively scarce.

Therefore, this study addresses the aforementioned issues and innovatively evaluates the effects of three types of TC exercise on improving the rate of change in BMD and reducing the risk of OP in middle-aged and older individuals at risk. It aims to provide evidence for the potential optimal exercise duration in TC exercise for the prevention and treatment of OP in the middle-aged and older population.

## Methods

### Research design

This investigation was structured as a randomized, double-blind controlled trial, executed with strict adherence to the ethical tenets delineated in the Declaration of Helsinki of the World Medical Association [22], and sanctioned by the Institutional Review Board or an autonomous ethics committee, empowered with the authority to oversee the design protocol. The research in this manuscript was approved by the Biomedical Ethics Committee of Qufu Normal University, No. 2022075, and the ethical materials state that the research is consented for publication, that the research is consented for publication (Additional file 1).

### Research subjects

#### Inclusion and exclusion criteria

This study recruited middle-aged and older individuals engaged in teaching activities in TC societies through random selection. Inclusion and exclusion criteria were established to limit variability and maintain subject baseline characteristics. Inclusion criteria included being between 55 and 65 years of age, voluntarily participating in TC club instruction, not being a manual laborer, being able to effectively perform the technical demands and loads of the movements during TC instruction, and having been assessed as "positive" by the ONE-MINUTE OSTEOPOROSIS RISK TEST (OMOST) [23]. Exclusion criteria were physical activity dysfunctional diseases, use of certain drugs for OP, malignant tumor bone metastases, multiple myeloma, septic myelitis, and spinal hemangioma, as well as primary or secondary hyperthyroidism.

#### Power analysis and sample size estimation

We conducted a power analysis and estimated the sample size using G*Power software (version 3.1.9.7) [24]. Based on previously published studies and expert recommendations, we determined an effect size of 0.5, a desired power of 0.80, and an alpha level of 0.05. Considering a potential attrition rate, we aimed to recruit a total of 60 participants for the study.

#### Subjects grouping

This study employed randomization for coding subjects who met inclusion criteria, utilizing stratified random grouping with SPSS 26.0 software. Personnel not involved in the study were responsible for coding subjects to four different groups, including 24TCCG (*n* = 15, 8 males/7 females), TCKFFG (*n* = 15, 7 males/8 females), TCSBG (*n* = 15, 8 males/7 females), and CG (*n* = 15, 7 males/8 females), but at a later stage, due to subject attrition the number of people in each group ended up being 24TCCG (*n* = 1324TCCG (*n* = 13, 7 males/6 females), TCKFFG (*n* = 12, 5 males/7 females), TCSBG (*n* = 11, 6 males/5 females), and CG (*n* = 13, 6 males/7 females). The intervention's purpose and design protocol were kept confidential from TC teachers and BMD measure participants to maintain blinding. However, blinding was not extended to the study subjects due to the instructional requirements of the TC exercise programs.

### TC exercise program and protocols

#### TC exercise program

In this study, we selected 24TCC, TCKFF, and TCSB as interventions for the participants, which are all related to Tai Chi (TC) but involve different exercise methods:

(i) 24TCC: It consists of 24 movements, including the basic actions and postures of TC, focusing primarily on the smoothness and coherence of movements, while emphasizing the fundamental principles of TC.

(ii) TCKFF: In TCKFF, the movements of TC are combined with the use of a fan, requiring practitioners to master the basic techniques of TC and learn how to perform various actions with the fan.

(iii) TCSB: This is a TC fitness tool typically composed of two metal balls connected by a rope. Practitioners rotate the balls in their hands to perform TC-style movements. The use of this tool helps improve hand–eye coordination, balance, and flexibility, with a focus on actions involving the hands and wrists.

#### Exercise protocols

Baseline characteristics of each subject group were recorded prior to experimentation, and homogeneity of the data was tested to ensure scientific and effective subsequent study. Mean and standard deviation data are presented in Table 1. According to the systematic review and meta-analysis conducted by Zhang et al. [18], TC exercise interventions have shown improvements in BMD among middle-aged and elderly individuals included in the studies. Considering the safety of the participants in our study, we referred to the protocol of this meta-analysis which was published in Chinese literature and made appropriate modifications based on it: The TC exercise program groups participated in a 60-min exercise regimen four times per week for 18 weeks (including a 2-week warm-up period and a 16-week formal training), including 15 min of warm-up and preparation activities before each formal training. Professional TC teachers instructed subjects in both basic movements and routines, as well as systematic teaching of specific programs. Exclusive TC program participation was required throughout the intervention, while subjects’ diet and living conditions remained constant. Interviews verified no major stressful events occurred during the exercise period, and excluded individuals were omitted from the treated sample. At the end of 16 weeks of formal training, 24TCCG, TCKFFG and TCSBG were evaluated.

**Table 1:** Baseline characteristics of the included subjects and data of each index before TC exercise

Baseline data	24TCCG (*n* = 13)	TCKFFG (*n* = 12)	TCSBG (*n* = 11)	CG (*n* = 13)	P-value
Demographic characteristics					
Years	60.0 ± 3.4	59.1 ± 2.7	58.6 ± 2.3	59.2 ± 2.7	> 0.05
Gender (males n/females n)	7/6	5/7	6/5	6/7	–
Educated at high school and above (n%)	2 (15.4%)	1 (8.3%)	3 (27.3%)	2 (15.4)	–
Educated at high school and below (n%)	11 (84.6%)	11 (91.7%)	8 (72.7%)	11 (84.6%)	–
Number and percentage of retirement cases (n%)	13 (100%)	12 (100%)	11 (100%)	13 (100%)	–
General health information					
Body weight (kg)	57.39 ± 2.98	56.91 ± 3.09	56.13 ± 2.17	56.11 ± 2.57	> 0.05
Height (cm)	171.22 ± 3.65	173.41 ± 4.17	172.76 ± 3.11	173.33 ± 2.44	> 0.05
BMI (kg/m^2^)	19.59 ± 0.87	18.93 ± 0.94	18.83 ± 1.14	18.69 ± 1.01	> 0.05
BMD (g/cm^2^)					
L_2_–L_4_ lumbar vertebrae	0.85 ± 0.09	0.82 ± 0.12	0.85 ± 0.17	0.79 ± 0.09	> 0.05
Ward's triangle	0.68 ± 0.07	0.72 ± 0.10	0.75 ± 0.13	0.69 ± 0.09	> 0.05
Femoral Neck	0.72 ± 0.08	0.78 ± 0.10	0.80 ± 0.14	0.74 ± 0.10	> 0.05
Greater trochanter	0.71 ± 0.08	0.75 ± 0.12	0.75 ± 0.14	0.75 ± 0.09	> 0.05

### Indicator measurements

This study utilized dual-energy X-ray absorptiometry (DXA) to measure BMD at four anatomical sites (L2-L4 lumbar vertebrae, Ward's triangle, femoral neck, and greater trochanter) over five consecutive noncardiac rate scans in g/cm^2^. The error coefficients for BMD measurements were 1–2% for lumbar vertebrae, femoral neck, and greater trochanter, and 2.5–5% for Ward's triangle [25]. The coefficient of variation was 1.1% for the lumbar vertebrae and 1.85% for the proximal femur [26]. BMD measurements were taken at 4-week intervals starting from the initiation of formal exercise for each program.

### Statistical analysis

#### Data entry

All BMD data and intervention results were subject to meticulous two-person cross-entry and verification. The intervention effect was assessed via the formula of (after intervention-before intervention)/before intervention × 100%, with percentages before and after intervention systematically observed. Given that BMD is a continuous variable in this context, we express it as mean ± standard difference (M ± SD) and determine 95% confidence intervals (CI).

#### Statistical analysis

Statistical analysis of data processing was performed using SPSS 26.0 software. Abnormal data and normal distribution were detected using the Shapiro–Wilk test in Explore, and any such data were systematically verified and eliminated. To determine the change rate of a given index between groups before and after the intervention, one-way analysis of variance (ANOVA) was utilized. Where *P* < 0.05, the statistical results were deemed significant, while *P* < 0.01 represented very significant differences. ANOVA was performed for each group, followed by post hoc multiple comparisons. Least significant difference (LSD) was used where *P* > 0.05 to determine data homogeneity of variance, while Tamhane 2 (T2) test was employed when *P* ≤ 0.05 indicated variance heterogeneity.

#### Image rendering

GraphPad Prism 8.0.2 software was utilized to generate graphical representations of BMD change rates in L2-L4, Ward's triangle, femoral neck, and greater trochanter regions before and after intervention in each group.

## Results

### Subjects were included in the completion condition

**Fig. 1:**
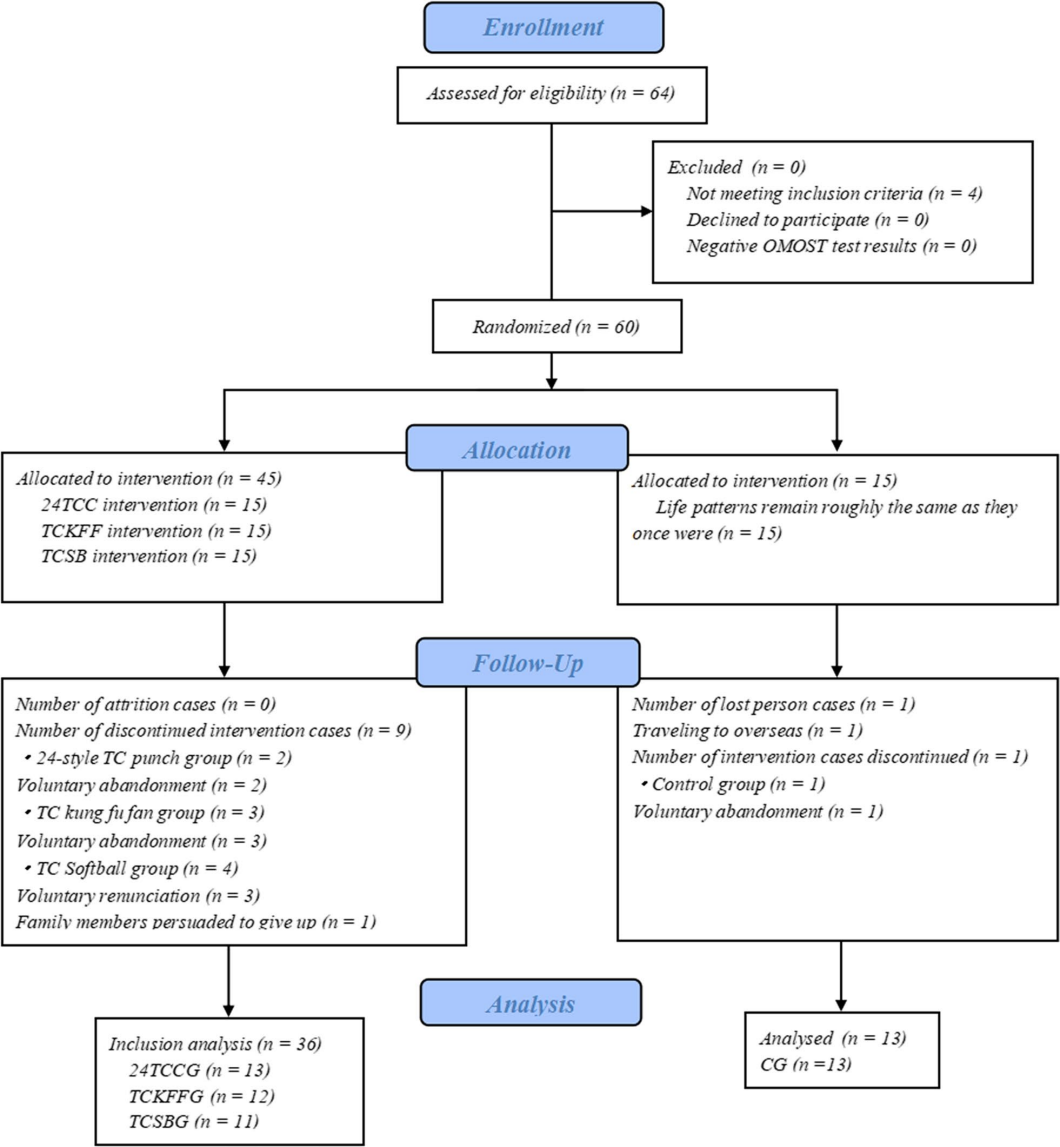
Flow program for detailed recruitment selection of subjects

From February to April in 2023, 64 subjects were recruited for this study, with 60 subjects identified as "positive" by OMOST after excluding 4 subjects who did not meet the inclusion criteria. These subjects were randomly allocated to 24TCCG, TCKFFG, TCSBG, and CG, each consisting of 15 cases. A total of 49 cases completed the trial after staff communication and consultation, with 11 cases lost. 24TCCG had 13 cases (86.7% compliance), TCKFFG had 12 patients (80% compliance), TCSBG had 11 patients (73.3% compliance), and CG had 13 patients (86.7% compliance). Details of the subject recruitment process are presented in Fig. 1.

### Baseline data and data after 16-week TC exercise intervention

#### Baseline data

Baseline data for each group are given in Table 1, including demographic characteristics (age, sex, education, and retirement) of the subjects, basic health information (weight, height, and BMI), and pre-intervention BMD at various sites (L2-L4 lumbar vertebrae, Ward's triangle, femoral neck, and greater trochanter). Prior to the experiment, the basic information and indices of each group were documented and assessed for homogeneity of previous data. It was observed that there were no significant differences in the baseline characteristics among the groups (*P* > 0.05), ensuring a scientifically rigorous implementation of the follow-up study.

#### Data of BMD changes

The recorded data on BMD changes are given in Table 2, including the rates of change for each individual TC exercise program intervention at baseline and on weeks 4, 8, 12, and 16 for specific skeletal regions such as L2-L4 lumbar vertebrae, Ward's triangle, femoral neck, and greater trochanter.

**Table 2:** Data of BMD changes after 16-week TC exercise intervention in each group

Measuring position	Intervention cycle	Rate of change in BMD (%)			
		24TCCG (*n* = 13)	TCKFFG (*n* = 12)	TCSBG (*n* = 11)	CG (*n* = 13)
L2-L4 lumbar vertebrae	Baseline	–	–	–	–
	4 weeks	− 0.012 ± 0.001	− 0.012 ± 0.002	− 0.012 ± 0.001	− 0.038 ± 0.004
	8 weeks	− 0.012 ± 0.001	− 0.025 ± 0.003	− 0.024 ± 0.004	− 0.061 ± 0.001
	12 weeks	− 0.024 ± 0.003	− 0.038 ± 0.005	− 0.036 ± 0.005	− 0.099 ± 0.014
	16 weeks	− 0.036 ± 0.004	− 0.051 ± 0.008	− 0.048 ± 0.007	− 0.014 ± 0.017
Ward's triangle	Baseline	–	–	–	–
	4 weeks	− 0.015 ± 0.002	− 0.014 ± 0.002	− 0.014 ± 0.002	− 0.044 ± 0.006
	8 weeks	− 0.017 ± 0.006	− 0.022 ± 0.008	− 0.026 ± 0.007	− 0.079 ± 0.017
	12 weeks	− 0.032 ± 0.007	− 0.036 ± 0.008	− 0.039 ± 0.009	− 0.012 ± 0.020
	16 weeks	− 0.047 ± 0.007	− 0.051 ± 0.010	− 0.050 ± 0.001	− 0.016 ± 0.024
Femoral neck	Baseline	–	–	–	–
	4 weeks	− 0.014 ± 0.002	− 0.013 ± 0.002	− 0.012 ± 0.002	− 0.041 ± 0.0051
	8 weeks	− 0.021 ± 0.007	− 0.020 ± 0.008	− 0.019 ± 0.006	− 0.075 ± 0.011
	12 weeks	− 0.035 ± 0.006	− 0.034 ± 0.009	− 0.032 ± 0.007	− 0.116 ± 0.015
	16 weeks	− 0.049 ± 0.007	− 0.046 ± 0.010	− 0.045 ± 0.008	− 0.157 ± 0.019
Greater trochanter	Baseline	–	–	–	–
	4 weeks	− 0.014 ± 0.001	− 0.014 ± 0.002	− 0.014 ± 0.002	− 0.040 ± 0.005
	8 weeks	− 0.019 ± 0.007	− 0.021 ± 0.008	− 0.022 ± 0.010	− 0.061 ± 0.009
	12 weeks	− 0.033 ± 0.007	− 0.034 ± 0.010	− 0.034 ± 0.012	− 0.101 ± 0.014
	16 weeks	− 0.047 ± 0.008	− 0.048 ± 0.011	− 0.049 ± 0.014	− 0.141 ± 0.019

### Results of statistical analysis

**Fig. 2:**
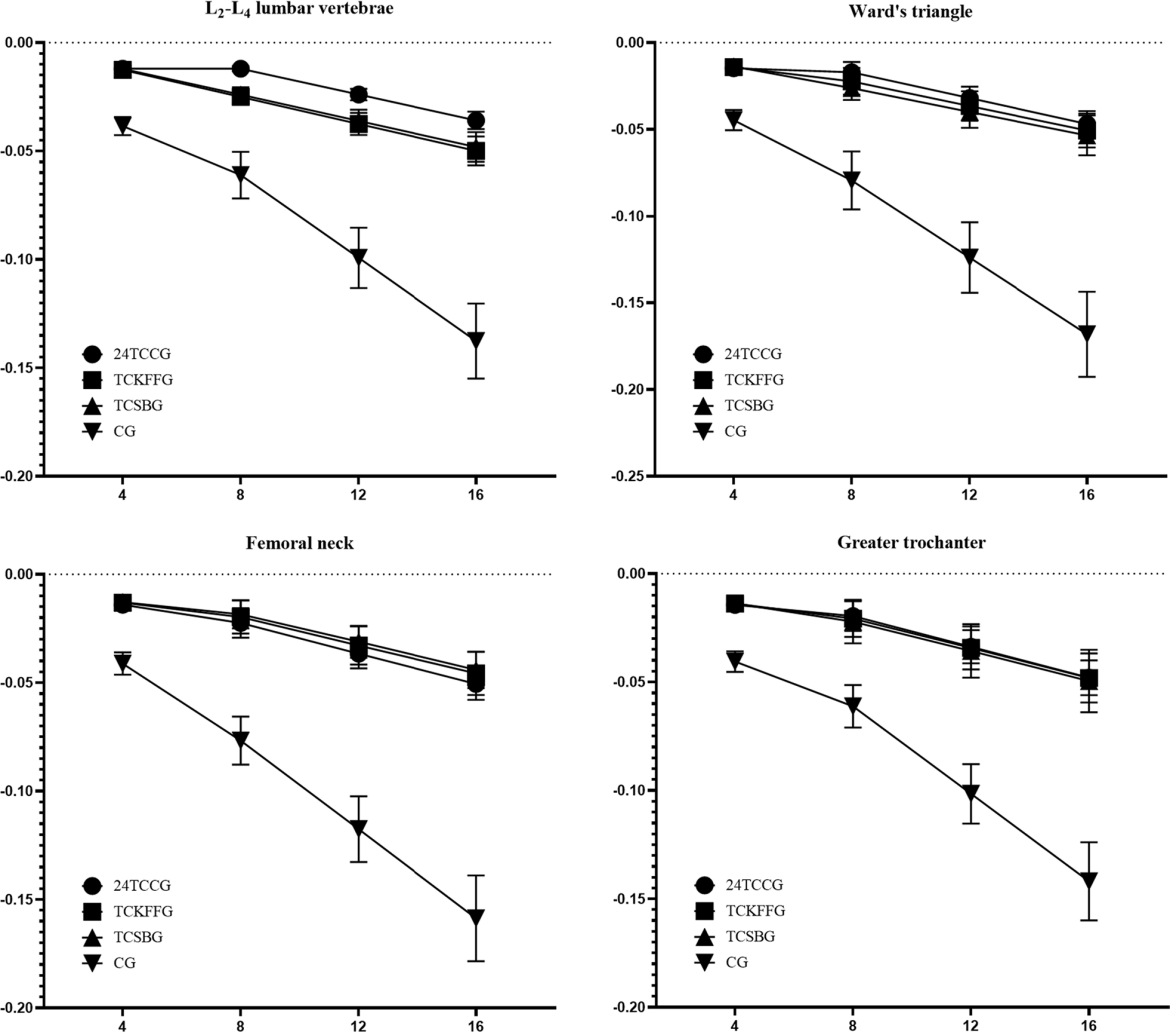
Trend plots that encapsulate the alterations in BMD subsequent to sixteen weeks of structured TC intervention across every study group. Note: The horizontal axis of each plot represents intervention duration (in weeks), whereas the vertical axis depicts the percentage rate of change in BMD (in %)

Figure 2 shows the trend plots of BMD changes in subjects before and after the intervention in each group.

#### L2-L4 lumbar vertebrae

During week 4, the comparison between 24TCCG and TCKFFG resulted in insignificant difference (*P* > 0.05) while a highly significant difference (*P* < 0.01) was observed between TCKFFG and CG. Furthermore, there was no significant difference between TCKFFG and TCSBG (*P* > 0.05) while a highly significant difference (*P* < 0.01) was observed between TCSBG and CG. Notably, highly significant differences (*P* < 0.01) were observed between TCKFFG and CG as well as TCSBG and CG. At weeks 8, 12, and 16, there were significant differences (*P* < 0.01) among 24TCCG and the other 3. Similarly, significant differences (*P* < 0.01) were observed between TCKFFG and CG while insignificant differences (*P* > 0.05) were observed between TCSBG and the other groups. Finally, significant differences (*P* < 0.01) were observed between TCSBG and CG during the same period.

#### Ward's triangle

At weeks 4, 12, and 16, no significant difference was observed between 24TCCG, TCKFFG, and TCSBG (*P* > 0.05). However, a highly significant difference was found in comparison to CG (*P* < 0.01). Additionally, no significant difference was detected between TCKFFG and TCSBG (*P* > 0.05), yet a highly significant difference was evident when compared to CG (*P* < 0.01). A highly significant difference was also observed between TCKFFG and TCSBG (*P* < 0.01) and between TCSBG and CG (*P* < 0.01). However, at week 8, a significant difference was found between 24TCCG and TCSBG (*P* < 0.05).

#### Femoral neck

At the 4th week, no significant difference was observed among 24TCCG, TCKFFG, and TCSBG (*P* > 0.05), while a highly significant difference was evident in comparison with CG (*P* < 0.01). Additionally, substantial differences were detected between TCKFFG and TCSBG as well as CG (both with *P* < 0.01). Following the 8th, 12th, and 16th week, no significant disparities were found among 24TCCG, TCKFFG, and TCSBG (*P* > 0.05), yet highly significant distinctions remained evident compared to CG (*P* < 0.01). Furthermore, no notable differences existed between TCKFFG and TCSBG (*P* > 0.05); however, highly significant differences persisted when compared to CG (*P* < 0.01). TCSBG and CG exhibited significant discrepancies (*P* < 0.01).

#### Greater trochanter

Following the assessments at the 4th, 8th, 12th, and 16th week intervals, there was no significant difference observed between 24TCCG, TCKFFG, and TCSBG (*P* > 0.05) while a highly significant difference was found in comparison to CG (*P* < 0.01). Additionally, there was no significant difference between TCKFFG and TCSBG (*P* > 0.05), yet a highly significant difference was noted when compared with CG (*P* < 0.01). A highly significant difference between TCSBG and CG was also evident (*P* < 0.01).

## Discussion

### Differences in the use of statistical methods between this study and other studies

Presently, considerable research is being conducted to investigate the influence of TC exercise on BMD in middle-aged and older populations. However, the employed statistical methodologies in these studies are diverse. For example, Chan et al. implemented the independent samples t-test to assess the impact of TC boxing exercise on BMD in postmenopausal women, as their study consisted of only 2 intervention groups [27]. This approach has also been utilized by other investigations in the field [28, 29]. In contrast to previous research, Wayne et al. employed the Wilcoxon rank-sum test, setting their study apart [30]. Recent investigations on the effects of various TC exercise programs on BMD in middle-aged and older adults [21, 31, 32] have predominantly utilized ANOVA. This approach has gained traction because a single comparison between two groups may not adequately represent the positive impact of diverse TC programs on BMD, given substantial variations in intensity, frequency, and duration. The current study also applied one-way ANOVA with post hoc multiple comparisons, albeit with several unique aspects. First, data preprocessing via Shapiro–Wilk in Explore was implemented to identify and remove anomalous data, thereby enhancing the study's scientific validity. Additionally, the Chi-square test was employed to ascertain the appropriate use of the LSD or T2 test in various situations, ultimately bolstering data analysis accuracy.

### Analysis of the results of this study

#### Controversial results on the effect of TC exercise on BMD

A comparative analysis of data from 24TCCG and CG demonstrated a significant enhancement in BMD at Ward's triangle and the femoral neck regions over time, paralleling findings for L2-L4 lumbar vertebrae and femoral neck BMD in TCKFFG and CG, along with L2-L4 lumbar vertebrae BMD and femoral neck BMD in TCSB and CG comparisons. The data imply that sustained TC exercise may considerably augment BMD. Nonetheless, the authors highlight the statistically significant improvements were transient, predominantly arising during the initial phase of the 16-week intervention and potentially diminishing subsequently. A meta-analysis investigating the impact of 24TCC on bone health among menopausal women revealed scarce evidence supporting its effectiveness, attributed to the weak, inconsistent evidence and negligible measured effects [17]. Consequently, additional thorough research is necessitated to ascertain the ideal duration and specific TC exercise regimens conducive to enhancing BMD in middle-aged and older adults, minimizing potential biases.

#### Differences in the effects of different TC exercise programs and times on BMD improvement

The present study revealed that each TC exercise program exhibited significant disparities in BMD changes across several skeletal regions including the L2-L4 lumbar vertebrae, Ward's triangle, femoral neck, and greater trochanter in comparison to CG, at various time points. These differences were particularly pronounced at select time points and may be attributable to the biomechanical characteristics of the body, as influenced by the movements performed during TC training. Such characteristics could conceivably impact skeletal muscle contraction and joint movement, ultimately impacting BMD. Our findings further suggest that the 24TCC exercise program is especially effective at mitigating BMD loss in the L2-L4 lumbar vertebrae over short-term training. Nonetheless, the training effects progressively converged with those of CG during the 8th, 12th, and 16th weeks of systematic training. This trend is consistent with the results of Cheng et al., who observed a more robust effect on BMD in the early phase of TC exercise, with a weaker effect in the later phases [21]. This suggests the existence of a situation in which the duration of TC exercise has different effects on the improvement of BMD in subjects.

The inherent adaptability of bone structure enables it to respond to mechanical stimuli, leading to the development of advantageous structural features in the mature skeleton through physical activity, such as enhanced cross-sectional area, BMD, and moment of inertia [33]. These characteristics collectively improve bone robustness and strength. The diverse skeletal adaptations to exercise are attributed to functional adaptation, the process where bone cells alter their structure in response to loading [34]. The comprehensive engagement of bodily motion subjects the skeletal framework to various external factors, including ground reaction forces and inertial properties, in addition to the internal stressors present within the skeletal musculature. Mechanosensitive cells within the bone, such as osteoblasts and osteoclasts, respond to atypical strain by initiating bone tissue resorption and new bone tissue formation. Thus, increased external force during total body-controlled exercise augments weaker bones by eliciting tissue strain and prompting osteogenesis. Initially, BMD loss is more pronounced than in CG, yet declines over time as the skeletal system strengthens.

The current study elucidates significant disparities in BMD changes in various skeletal regions, including L2-L4 lumbar vertebrae, Ward's triangle, femoral neck, and greater trochanter, during TC exercise programs compared to CG. These disparities were notably prominent at specific time points, potentially attributable to biomechanical characteristics of the body influenced by TC exercise, which could affect skeletal muscle contraction, joint movement, and ultimately, BMD. 24TCC demonstrates particular efficacy in mitigating BMD loss in L2-L4 lumbar vertebrae during short-term training, but training effects converge with those of CG over longer durations (i.e., 8th, 12th, and 16th weeks). This temporal variation in TC exercise efficacy on BMD improvement aligns with Cheng et al. [20]. Indeed, bone structure is inherently responsive to mechanical environments, and physical activity can foster the development of advantageous structural attributes in the mature skeleton, such as increased cross-sectional area, BMD, and moment of inertia [29]. These skeletal adaptations to exercise derive from functional adaptation, a process wherein bone cells modify their structure in response to the load. During total body-controlled exercise, both external (e.g., ground reaction, inertia) and internal (e.g., skeletal muscle) forces generate stress on the skeletal system, determining bone tissue deformation and subsequent mechanical strain. Mechanosensitive cells within the bone (e.g., osteoblasts, osteoclasts) detect atypical strain and trigger an adaptive response resulting in bone tissue resorption and generation [34]. Consequently, heightened external force during total body-controlled exercise fortifies weaker bones through increased tissue strain, stimulating osteogenesis. BMD loss is initially higher compared to CG, but diminishes over time as the skeletal system strengthens.

#### The potential impact of gender disparities on the trend in BMD changes

In this study, the number of males and females varies among different groups. Based on the existing research, females are more susceptible to OP due to hormonal differences, changes in menopausal hormones, and variations in peak bone mass [35]. Depending on whether each group has a higher proportion of females or males, it can be inferred that this may exert a certain influence on the BMD change rates within each group. In the TCKFFG and CG, females slightly outnumber males, and as females are more prone to OP, it can be hypothesized that the BMD change rates among females in these groups may be relatively higher, potentially resulting in larger BMD change trends in these groups. However, due to the relatively small sample sizes, these findings require validation in larger-scale studies. These discoveries suggest that gender disparities may play a role in the influence of physical exercise on BMD changes. Future research can delve deeper into the effects of gender factors on the improvement of BMD through physical exercise by utilizing larger sample sizes and achieving a more balanced gender distribution.

### Potential reasons for the decreasing trend in the rate of change of BMD in the subjects of this study

The present study utilized Plot trend analysis of BMD data by site to reveal a decrease in BMD in subjects, possibly due to the risk of OP. It has been suggested that a negative correlation exists between BMI and the risk of OP prevalence, with a higher risk of OP prevalence in subjects with lower BMI [36]. Though the included subjects had normal BMIs according to World Health Organization (WHO) [37], they were still at risk of OP due to being on the lower end of the normal range. OP risk is characterized by bone marrow presence and a reduction in BMD. The observed decreasing trend in BMD indicators in the studied subjects can plausibly be explained by this [38]. However, the Plot trend analysis also showed that the BMD of the 3 groups with TC exercise decreased at a slower rate compared to CG, indicating that TC exercise had a positive impact on improving BMD.

### Potential reasons for better improvement in BMD with 24TCC compared to other TC exercise programs

In our study, we observed a better effect of 24TCC on BMD improvement, which may be attributed to a combination of biomechanical, physiological, and potential biochemical factors. Several potential mechanisms can be proposed:

#### Advantage of movement characteristics

The superior effect of 24TCC and other TC exercise programs on BMD improvement may be attributed to their movement characteristics. Firstly, the 24TCC program consists of 24 different movements, focusing on the smoothness and continuity of the overall movements. These movements involve various joint motions and provide more diverse and intense mechanical stimuli to the skeletal system compared to other TC programs [39]. The greater mechanical stimuli produced by the specific movements in 24TCC may result in more significant adaptations in bone tissue, ultimately enhancing BMD. Additionally, the 24TCC program combines smooth movements, weight shifting, and resistance exercises. In particular, the resistance exercises, which apply mechanical stress to the bones, have been shown to stimulate bone remodeling and increase BMD. The weighted nature of some movements in 24TCC, such as single-leg standing and transitions between different stances, may contribute to the observed positive effects on BMD [39]. Furthermore, the 24TCC program emphasizes the basic principles of TC exercise, including correct body posture, mindful movement, and coordinated breathing [15]. These principles are crucial for optimizing the transfer of strength and energy in the body during 24TCC practice. Driven by these principles, the precise execution of 24TCC movements can enhance the efficiency of force transmission in the musculoskeletal system, thereby improving bone health.

#### Biomechanical characteristics

The superiority of 24TCC in improving BMD can be attributed to specific movements and biomechanical characteristics inherent in the program. The 24TCC training program consists of 24 different movements involving various joint motions, including weight shifting, rotations, and weighted postures. Some of these movements, such as single-leg standing and transitions between different stances, require a higher level of balance and strength compared to TCKFF and TCSB, which may impose greater mechanical loads on the skeletal system [40]. The weighted nature of some movements in 24TCC, such as horse stance and high pat on horse, may stimulate bone remodeling and increase BMD. It has been demonstrated that weight-bearing exercise can subject the bones to mechanical loading, leading to increased bone density and beneficial effects on bone health [40]. By incorporating these weight-bearing movements, 24TCC can provide more specific and targeted stimulation to the bones, resulting in a more pronounced improvement in BMD.

#### Physiological and biochemical mechanisms:

The superiority of 24TCC in improving BMD may involve physiological mechanisms. The execution of movements in 24TCC involves the integration of body alignment, breathing techniques, and mindful coordination. The precise execution of movements and the combination of mental focus may enhance the activation of neuromuscular activity and recruitment of skeletal muscle groups, potentially stimulating bone metabolism and enhancing BMD. Furthermore, previous research has suggested that specific movements in 24TCC, such as brush knee twist step and wave hands like clouds, generate intensity and impact that can promote the release of growth factors and hormones associated with bone remodeling. These signaling molecules, including IGF-1 [41] and TGF-β [42], have been shown to have positive effects on bone health and promote osteoblast function and bone formation.

### Potential mechanisms for TC exercise to improve BMD

#### Physical mechanisms

This study employed a RCT to investigate the effects of TC exercise on the prevention and treatment of OP in middle-aged and elderly individuals. While the results provide insight into the impact of TC exercise on BMD improvement, it is imperative to elucidate the underlying mechanism, which potentially involves mechanical loading-induced osteogenesis [43]. In this population, age-related physiological deterioration contributes to bone metabolism disturbances and imbalanced bone resorption and formation, resulting in increased bone mineral loss, heightened susceptibility to OP, and fractures. Additionally, reduced physical mobility, muscle strength, and body balance exacerbate their limited physical activity capacity [44]. Evidence suggests that exercise offers mechanical stimulation to joint tissues and bones [45], and that exercise-generated mechanical stress stimulation plays a crucial role in osteoblast differentiation, mineralization, and BMD maintenance [46].

The underlying mechanism of how TC exercise enhances BMD in middle-aged and elderly individuals remains elusive. Nonetheless, it is established that the deterioration of bone metabolism in adults can result in an imbalance between bone resorption and bone formation, leading to loss of bone mineral [47]. This dysfunction subsequently causes alterations in bone morphology, including changes in DNA and collagen synthesis, which contribute to a decline in BMD. While exercise alone cannot fully counteract the reduction in bone mineral content, it has notable effects on bone blood circulation and can mitigate bone mineral loss, actively foster bone cell formation, and thus play a crucial role in the prevention of OP [31]. Moderate exercise is known to significantly elevate overall BMD, modulate bone metabolism, and moderately increase BMD throughout the body. Additionally, TC exercise generates muscle contractions that directly or indirectly impact bone, inducing alterations in bone voltage and subsequently stimulating osteoblast formation. These effects not only help maintain or increase bone density but also enhance bone elasticity and resilience to bending, compression, and twisting forces. Furthermore, the distinctive movements and gentle oscillations of TC can massage the periosteum, promoting blood supply to bone tissue and facilitating the uptake of bone nutrients [48]. As an aerobic exercise, TC also fosters long-term improvements in muscle strength, coordination, and balance, which aid in the restoration of bone structure and mass, stimulate osteoblast activity, and ultimately increase BMD.

#### Biochemical mechanisms

The engagement in TC exercise has been demonstrated to generate mechanical stress on the skeletal system, subsequently stabilizing bone metabolism and enhancing BMD. Notably, this exertion may also potentially elicit hormonal, cytokine, noncoding RNA, and signaling pathway responses, thereby fostering osteogenesis.

TC exercise may induce hormones and cytokines that promote osteogenesis:

The theoretical hypothesis proposes that performing TC exercise can stimulate the production of hormones and cytokines, ultimately leading to the promotion of osteogenesis [44, 49]. Previous research has demonstrated that exercise can effectively modulate various hormones, including estrogen, parathyroid hormone, and glucocorticoids, all of which are known to play a crucial role in bone metabolism and remodeling. For instance, Bentz et al. proposed that exercise can enhance the secretion of estrogen (estradiol) in pre-menopausal women, partially replicating the effects of hormone replacement therapy for OP [49]. In addition, other studies have indicated that exercise may result in elevated levels of serum estradiol (E2) [50]. Moreover, TC exercise often resembles resistance training, which has been suggested by Sato et al. to potentially increase serum testosterone levels in older men and subsequently decrease the loss of BMD [50]. Furthermore, exercise appears to have an impact on the secretion of pro-inflammatory cytokines that are involved in bone resorption, such as IL-1, IL-6, and TNF-α. Through exercise, the secretion of these cytokines is reduced, while the absorption of protective cytokines including IL-2, IL-10, IL-12, IL-13, IL-18, and IFN is enhanced [51]. Additionally, there is evidence suggesting that the variation at the translation initiation site of the vitamin D receptor gene is associated with the response to OP [52].

TC exercise may induce noncoding RNAs to promote osteogenesis:

Noncoding RNAs, including siRNA, microRNA, lncRNA, and circRNA, have been identified as important modulators of bone metabolism. Specifically, noncoding RNAs are implicated in regulating osteoblast and osteoclast proliferation and differentiation, and TC exercise has been shown to stimulate the production of these molecules [53]. TC exercise generates mechanical stress, which in turn can activate microRNAs that may have therapeutic implications for skeletal-related diseases such as OP [54, 55]. Studies have demonstrated that mechanical stress upregulates the expression of miRNA-191 and miRNA-3070a, while downregulating miRNA-218 and miRNA-33; these miRNAs have been found to be involved in the regulation of osteoblast differentiation [56]. Additionally, TC exercise may promote osteogenesis by targeting miRNA-103a, which is downregulated in response to mechanical stress [57]. Finally, it has been discovered that mechanical stress can also modulate the expression of long-stranded noncoding RNAs, further promoting osteogenesis [58].

TC exercise may induce relevant signaling pathways to promote osteogenesis:

The primary signaling pathways that play a crucial role in bone metabolism include the Wnt/β-catenin, BMP, OPG/RANKL/RANK, and Notch pathways [59]. Research has shown that exercise has the ability to enhance bone remodeling by upregulating these pathways. This leads to the differentiation of osteoblasts, the inhibition of osteoclast activity, and improved bone remodeling, ultimately preventing OP [54–60]. Moreover, exercise can also exert mechanical stress on bones, which triggers the activation of additional pathways such as PPERK-eIF2α-ATF4, mTORC2-Akt-GSK3β, and PI3K/Akt/GSK-3β/β-catenin. These pathways further promote osteogenesis [60]. Therefore, it is possible that exercise using TC (traditional Chinese) exercises may promote osteogenesis by inducing mechanical stress and modulating these signaling pathways, leading to improved BMD and prevention of OP.

Additionally, factors such as bone-specific alkaline phosphatase (BLP), procollagen type I N-terminal propeptide (PINP), β C-terminal telopeptide of type I collagen (BCTX), and N-terminal telopeptide of type I collagen (NTX) have been shown to impact bone metabolism and remodeling. Exercise has been demonstrated to modulate these factors as well [61, 62].

TC exercise improves bone metabolism by enhancing fat content

Existing research suggests that physical activity may influence bone metabolism by improving fat content [63]. Studies in this regard have found that physical activity can reduce the accumulation of adipose tissue, thereby alleviating the degree of obesity [63]. Obesity is one of the risk factors for bone metabolism disorders such as OP and is associated with a decrease in bone density and the occurrence of OP [63]. Therefore, TC exercise may contribute to the enhancement of bone metabolism and the improvement of BMD by reducing fat content. However, it is worth noting that the subjects included in this study had a lower BMI, excluding the potential impact of obesity, which may result in some limitations in the applicability of this mechanism to the subjects in this study.

### Insights from TC exercise in traditional Chinese exercise control OP

Research has demonstrated the high prevalence of OP in our country [64]. Studies conducted in recent years have confirmed the beneficial impact of exercise on enhancing OP, as exercising moderately can increase skeletal muscle flexibility, balance function, bone strength, and structural health, while decelerating BMD loss. Nuti et al.'s OP rehabilitation guidelines recommend engaging in moderate exercise to promote osteogenesis, regulate the endocrine system, elevate estrogen levels, and help prevent BMD loss, thus facilitating OP management [65]. Furthermore, the expert consensus guidelines of Izquierdo et al. argue that structured exercise is an important preventative strategy for chronic diseases including OP, and enhances functionality throughout the aging process [66]. Traditional Chinese exercises, such as TC, combine aerobic exercise with traditional Chinese medicine healthcare, promoting physical health by integrating physical activity, respiration, and psychological regulation. Long-term participation in TC exercises can lead to a reduction in bone loss, bone pain, and OP risk [67].

### Advantages and limitations

There exist several advantages to the present study: (i) Subjects in this study underwent OMOST and tested positive, thereby mitigating the error in baseline characteristics and selection bias, resulting in increased credibility of the RCT outcomes. (ii) In the statistical analysis of this study's RCT data, the exclusion of baseline characteristics and measured indexes in the attrition population led to a reduction in result dispersion and an increase in result credibility. (iii) By rigorously establishing inclusion and exclusion criteria for subjects, bias in subject selection was reduced and the credibility of study results was enhanced; the implementation of such criteria contributed to reducing bias further and increasing the reliability of the RCT outcomes.

There exist certain limitations to the present study: (i) Sample size and attrition: The sample size in this study was limited, and attrition led to a reduction in the number of participants. Future studies should aim for larger sample sizes to increase statistical power and minimize the impact of attrition on the results. (ii) Baseline characteristics: Although efforts were made to ensure comparable baseline characteristics, there may still be variations among individuals. Future studies could consider stratifying participants based on baseline characteristics to reduce confounding factors and enhance result validity. (iii) Adherence to TC exercise: It is possible that the adherence to TC exercise interventions varied among participants, potentially affecting the outcomes. Future studies should consider monitoring adherence rates and potential influencing factors to better understand the impact of adherence on the results. (iv) Generalizability: The participants in this study were self-selected, which may limit the generalizability of the findings. Future studies could consider recruiting participants from a wider range of populations to improve the external validity of the results. (v) Outcome measurements: Although DXA was used to measure BMD in this study, there is a margin of error associated with this method. Future studies could consider using additional measurement techniques or incorporating multiple outcome measures to provide a more comprehensive evaluation of BMD.

### Suggestions

(i) Larger sample size: Conduct studies with a larger sample size to increase statistical power and enhance the generalizability of the findings. (ii) Long-term interventions: Implement longer intervention periods to investigate the effects of TC exercise programs on BMD over extended durations. (iii) Stratification based on baseline characteristics: Ensure baseline characteristic homogeneity by stratifying participants to minimize potential confounding factors. (iv) Adherence monitoring: Monitor participants' adherence to TC exercise interventions and investigate factors influencing adherence rates. (v) Diverse population inclusion: Expand the study population to include individuals with different demographic characteristics to improve external validity. (vi) Alternative outcome measures: Consider using additional measurement techniques or incorporating multiple outcome measures to provide a comprehensive evaluation of BMD and its changes.

## Conclusion

In conclusion, all 3 TC exercise programs yielded comparable positive outcomes on BMD in the femoral neck and greater trochanter. Nevertheless, 24TCC, the long-term (≥ 8 weeks) TC exercise intervention, exhibited superior improvements in BMD at L2-L4 lumbar vertebrae when compared to the other programs. Furthermore, 24TCC was more effective than TCSB in enhancing BMD at Ward's triangle in 8 weeks. Lastly, the short-term (≤ 4 weeks) TC exercise intervention, TCKFF, was found to be more efficacious than TCSB in augmenting BMD at the femoral neck region. This study presents proof supporting the efficiency of TC exercise in enhancing BMD and inhibiting OP in individuals of middle-aged and elderly who are at a higher risk.

### Supplementary Information

**Additional file 1**. Ethical Materials.

## Data Availability

The datasets used in this study are available upon request from the first or corresponding author. Access to the data is subject to any applicable ethical and legal approvals.
