# Supplementary material for: Effect of different types of Tai Chi exercise programs on the rate of change in bone mineral density in middle-aged adults at risk of osteoporosis: a randomized controlled trial
Source: J Orthop Surg Res. 2023 Dec 11;18:949. doi: 10.1186/s13018-023-04324-0 (PMC10712169; doi:10.1186/s13018-023-04324-0)
Supplement: Supplementary file 1 — Additional file 1. Ethical Materials. [file 13018_2023_4324_MOESM1_ESM.pdf]

Qufu Normal University Biomedical Ethics Approval Document

No. (Nọ): 2022075-

Lei Zhu The type of project applied for (submitted) is as follows: Sports Science The project (thesis) "**Effect of different types of Tai Chi exercise programs on the rate of change in bone mineral density in middle-aged adults at risk of osteoporosis: a randomized controlled trial**", which was examined by the Bio-medical Ethics Committee, complied with the ethical principles, and agreed to be declared *(submitted)*.

Biomedical Ethics Committee of Qufu Normal University

Signature of the Director (Deputy Director) of the Ethics Committee

February 20, 2022
